# Prediction of sleep quality among university students after analyzing lifestyles, sports habits, and mental health

**DOI:** 10.3389/fpsyt.2022.927619

**Published:** 2022-08-04

**Authors:** Lirong Zhang, Hua Zheng, Min Yi, Ying Zhang, Guoliang Cai, Changqing Li, Liang Zhao

**Affiliations:** ^1^Department of Physical Education, Xiamen University of Technology, Xiamen, China; ^2^College of Physical Education and Health Sciences, Chongqing Normal University, Chongqing, China; ^3^Institute of Medical Information, Chinese Academy of Medical Sciences, Peking Union Medical College, Beijing, China; ^4^Department of Physical Education, North China University of Water Resources and Electric Power, Zhengzhou, China; ^5^College of Sports Human Science, Harbin Sport University, Harbin, China

**Keywords:** quality of sleep, Pittsburgh Sleep Quality Index, university students, depression anxiety stress scales, prediction model

## Abstract

The aim of this study was to develop and validate a prediction model to evaluate the risk of poor sleep quality. We performed a cross-sectional study and enrolled 1,928 college students from five universities between September and November 2021. The quality of sleep was evaluated using the Chinese version of the Pittsburgh Sleep Quality Index (PSQI). Participants were divided into a training (*n* = 1,555) group and a validation (*n* = 373) group. The training group was used to establish the model, and the validation group was used to validate the predictive effectiveness of the model. The risk classification of all participants was performed based on the optimal threshold of the model. Of all enrolled participants, 45.07% (869/1,928) had poor sleep quality (PSQI score ≧ 6 points). Multivariate analysis showed that factors such as older age, a higher grade, previous smoking, drinking, midday rest, chronic disease, anxiety, and stress were significantly associated with a higher rate of poor sleep quality, while preference for vegetables was significantly associated with better sleep quality, and all these variables were included to develop the prediction model. The area under the curve (AUC) was 0.765 [95% confidence interval (CI): 0.742–0.789] in the training group and 0.715 (95% CI: 0.664–0.766) in the validation group. Corresponding discrimination slopes were 0.207 and 0.167, respectively, and Brier scores were 0.195 and 0.221, respectively. Calibration curves showed favorable matched consistency between the predicted and actual probability of poor sleep quality in both groups. Based on the optimal threshold, the actual probability of poor sleep quality was 29.03% (317/1,092) in the low-risk group and 66.03% (552/836) in the high-risk group (*P* < 0.001). A nomogram was presented to calculate the probability of poor sleep quality to promote the applicationof the model. The prediction model can be a helpful tool to stratify sleep quality, especially among university students. Some intervention measures or preventive strategies to quit smoking and drinking, eat more vegetables, avoid midday rest, treat chronic disease, and alleviate anxiety and stress may be considerably beneficial in improving sleep quality.

## Introduction

Poor sleep quality is one of the severe health issues and is prevailing among teenagers who are vulnerable to the adverse effect of unsatisfactory sleep quality because of social or environmental shocks and their inclinations to stay up late (1). According to the available literature, up to 31.00–65.00% of university students had poor sleep quality (2–5). In particular, sleep problems can lead to various adverse outcomes, including a decreased academic performance (6), an increased risk for insomnia, high blood pressure (7), cognitive impairment, decreased quality of life, negative mental health condition (8), and even suicide ideation (9).

Several risk characteristics, such as grade (10, 11), stress (4, 12), physical activity (11), alcohol use (2), substance abuse (13), and severity of smartphone use (14, 15), associated with poor sleep quality were identified. These variables were able to guide doctors to roughly screen patients at a high risk of poor sleep quality. However, these variables could not accurately calculate the risk probability of having poor sleep quality, and thus early detection was difficult. Accordingly, this might result in not only inadequate diagnosis and therapeutic interventions but also overtreatment or improper administration of hypnosedatives (16). Thus, a valid tool that was able to cluster sleep quality was warranted before we could perform individualized healthcare. In addition, a model to evaluate the probability of poor sleep quality would be of great help to early detect the status, stratify risk, and treat this negative outcome individually. The currently available prediction models to predict the quality of sleep were particularly designed for elderly patients (16), rescuers (17), and caregivers (18). Nonetheless, data on prediction models for calculating the risk of poor sleep quality were extremely limited, especially among university students.

Therefore, the aim of this study was to investigate potential risk factors associated with poor sleep quality and further develop and validate a prediction model to measure the risk of poor sleep quality, especially among university students. This study hypothesized that significant variables associated with poor sleep quality could be identified and used to create a nomogram, which would accurately and individually evaluate the probability of suffering from poor sleep quality, especially among university students.

## Materials and methods

### Participants and study design

This study conducted a cross-sectional survey and analyzed 2,003 college students from five universities [Chongqing Normal University (Chongqing), Xiamen University of Technology (Xiamen), Harbin Sport University (Harbin), Sichuan Normal University (Chengdu), and North China University of Water Resources and Electric Power (Zhengzhou)] between September and November 2021 in China. University students voluntarily responded and completed the survey. The survey was distributed *via* instant communication tools, such as telephone messages and WeChat software, through a non-probability snowball sampling strategy (19). The survey consisted of the Student’s basic information, lifestyles, comorbidities, mental health status, and evaluation of sleep quality. In addition, coronavirus disease 2019 (COVID-19) sporadic outbreaks were also reported by participants according to the real status of the great pandemic in their living city.

**FIGURE 1:**
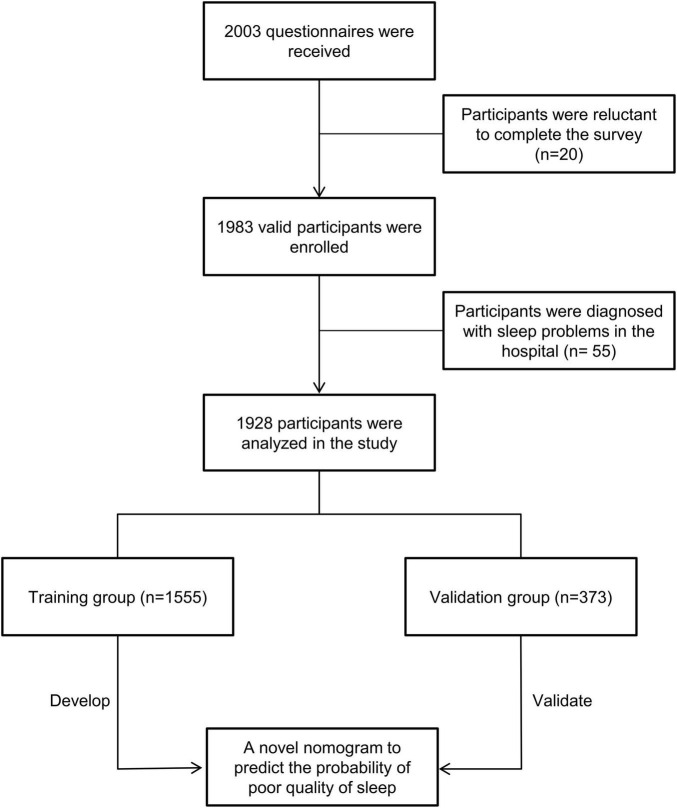
Patient’s flowchart and study design. Based on inclusive and exclusive criteria, a total of 1,928 participants were enrolled and divided into a training group (*n* = 1,555) and a validation (*n* = 373) group.

University students who were previously diagnosed with sleep problems in the hospital or did not want to complete the survey were not included in the analysis. Figure 1 depicts the flowchart of participants, and a total of 1,928 university students were enrolled. To maximize data utilization, the majority of participants were used to develop the model. Thus, participants from the first four centers (*n* = 1,555) were employed as the training group, which was used to establish the model to predict sleep quality; participants from North China University of Water Resources and Electric Power (*n* = 373) were employed as the validation group, which was used to validate the predictive effectiveness of the model.

This study was approved by the Academic Committee and Ethics Board of the Xiamen University of Technology (no. 202001). Formal consent was obtained from all participants, and their identified personal information was not collected. This study was conducted in accordance with the Declaration of Helsinki.

### Evaluation of sleep quality

The quality of sleep was evaluated using the Chinese version of the Pittsburgh Sleep Quality Index (PSQI). It was widely used among the Chinese population (20). It has 19 items and captures seven domains, namely, the subjective sleep quality, the sleep latency, the sleep duration, the habitual sleep efficiency, the sleep disturbances, the use of sleeping drugs, and the daytime dysfunction (21). Each domain has a score of 0–3, and the total score of PSQI, ranging from 0 to 21, was the combination of the seven domains. The PSQI score was the indicator variable to cluster participants with poor or good sleep quality. In detail, poor sleep quality was defined as participants with a total PSQI score of six points or above, while good sleep quality was defined as participants with a total PSQI score of less than six points (16, 22).

### Potential risk features

The study evaluated 22 potential risk features for their ability to predict poor sleep quality, including basic information [gender, age (years), grade, and marital status], hobbies (smoking and drinking), living habits [having a habit of midday rest, monthly expense (¥), preference to low salt and fat food, preference to oil food, preference to barbecue, preference to red meat, preference to vegetable, and preference to fruit], sports habits [sedentary time per day (hours), frequency of sports per week, and sports type], comorbidities (chronic disease), COVID-19 sporadic outbreak in the local city, and mental health status (depression, anxiety, and stress).

All the above variables were reported by participants based on their actual conditions. Chronic disease was defined as participants with a diagnosis of chronic disease in a hospital, such as hypertension, diabetes, congenital heart disease, chronic kidney disease, chronic lung disease, chronic liver disease, and others. The frequency of sports per week was the number of sports that participants did each week, and the total time of each workout should be a minimum of 30 min. A COVID-19 sporadic outbreak was defined as at least one patient being diagnosed with COVID-19 during the last 2 weeks in the participant’s local city. Depression, anxiety, and stress scores were evaluated using Depression Anxiety Stress Scales-21 (DASS-21) (23).

### Establishment of the model

Participants in the training group were used to establish the model, and significant variables identified by the multiple logistic regression analysis were included in the model. The model was presented in the format of a nomogram, and the “regplot” R package was used to create the nomogram to calculate the risk probability of poor sleep quality and to promote the application of the model.

### Validation of the model

Internal validation of the model was performed in both the training and validation groups. The predictive effectiveness of the model was evaluated using discrimination and calibration. In the study, discriminative ability was the model’s capability to identify participants with poor sleep quality and those without poor sleep quality, and this metric mainly consisted of area under the curve (AUC) and discrimination slope. The calibrating ability was the model’s capability to confirm the homogeneity between the actual and predicted probability of having poor sleep quality. In this study, calibrating ability was evaluated using the calibration curve and Brier score (24). A calibration curve was plotted using the Bootstrap method after applying 500 iterations. A Brier score is the mean squared difference between a patient’s predicted probability and actual status (1 or 0 depending on whether the event is positive or negative). If the Brier score approaches 0.0, it usually indicates a perfect prediction. Decision curve analysis was used to assess the clinical usefulness of the model. In addition, the model’s accuracy, sensitivity, specificity, negative predictive value (NPV), positive predictive value (PPV), precision, recall, and Youden index were also evaluated in the study.

### Statistical analysis

Participants’ sociodemographic characteristics were presented in proportions or median and interquartile range (IQR). According to the optimal threshold, all participants were divided into two risk groups, namely, a low-risk group (participants with a predicted probability of less than the threshold) and a high-risk group (participants with a predicted probability of threshold or above). The Chi-square test was used to compare the difference in the actual probability of poor sleep quality between the low-risk group and the high-risk group. A *P*-value of less than 0.05 was regarded as statistical significance (two-sided tests). All statistical analyses and data visualization were performed using R programming language software (version 4.1.2).

## Results

### Participant’s demographics, lifestyles, and mental health

Among the total participants, the majority of them were female (55.13%, 1,063/1,928) and the median age was 19.00 (19.00, 20.00) years. The majority of participants were sophomore (47.10%, 908/1,928) and single (76.56%, 1,476/1,928). Only a small fraction of participants were current smoker (4.93%, 95/1,928) or drinker (13.69%, 264/1,928). Of all the included participants, 78.89% (1,521/1,928) had a habit of midday rest, and 78.89% (1,521/1,928) had a monthly expense of less than 2,000 ¥. Furthermore, less than 5.00% of participants had previously been diagnosed and hospitalized for chronic disease. Notably, 48.60% (937/1,928) of participants were living in a city that had COVID-19 sporadic outbreaks. Based on the evaluation of DASS-21, the median depression, anxiety, and stress scores were 4.00 (0.00 10.00), 4.00 (0.00 10.00), and 6.00 (0.00 12.00), respectively, which indicated that the majority of participants were generally in a healthy mental condition. But, sleep quality was not satisfactory among those participants, and 45.07% (869/1,928) of them suffered from poor sleep quality (PSQI score ≧ 6). Furthermore, Table 1 shows more details about the participant’s food preferences and sports habits.

**TABLE 1:** Student’s demographics, living and sport habits, and mental health.

Characteristics	Students (*n* = 1,928)
Gender	
Male	44.87% (865/1,928)
Female	55.13% (1,063/1,928)
Age [median (IQR), years]	19.00 (19.00, 20.00)
Grade	
First year	24.27% (468/1,928)
Second year	47.10% (908/1,928)
Third year	16.65% (321/1,928)
Fourth year	11.15% (215/1,928)
Delayed graduation	0.83% (16/1,928)
Marital status	
Single	76.56% (1,476/1,928)
Dating	22.61% (436/1,928)
Married	0.83% (16/1,928)
Smoking	
No	92.12% (1,776/1,928)
Abstain from smoking	2.96% (57/1,928)
Yes	4.93% (95/1,928)
Drinking	
No	81.64% (1,574/1,928)
Abstain from drinking	4.67% (90/1,928)
Yes	13.69% (264/1,928)
Having a habit of midday rest	
Yes	78.89% (1,521/1,928)
No	21.11% (407/1,928)
Monthly expense (¥)	
<2,000	78.89% (1,521/1,928)
≧2,000 and < 5,000	20.23% (390/1,928)
≧5,000 and < 10,000	0.41% (8/1,928)
≧10,000	0.47% (9/1,928)
Chronic disease	
Yes	4.10% (79/1,928)
No	95.90% (1,849/1,928)
Preference to low salt and fat food	
Yes	30.60% (590/1,928)
No	69.40% (1,338/1,928)
Preference to oil food	
Yes	25.83% (498/1,928)
No	74.17% (1,430/1,928)
Preference to barbecue	
Yes	28.99% (559/1,928)
No	71.01% (1,369/1,928)
Preference to red meat	
Yes	66.65% (1,285/1,928)
No	33.35% (643/1,928)
Preference to vegetable	
Yes	49.17% (948/1,928)
No	50.83% (980/1,928)
Preference to fruit	
Yes	57.47% (1,108/1,928)
No	42.53% (820/1,928)
Sedentary time (hours)	
<1	5.13% (99/1,928)
≧1 and < 3	18.36% (354/1,928)
≧3 and < 6	33.45% (645/1,928)
≧6	43.05% (830/1,928)
Frequency of sports per week	
0	21.27% (410/1,928)
1−2	36.41% (702/1,928)
3−4	21.06% (406/1,928)
≧5	21.27% (410/1,928)
Sport type	
None	21.27% (410/1,928)
Aerobic exercise	44.29% (854/1,928)
A middle between aerobic and anaerobic exercise	23.24% (448/1,928)
Anaerobic exercise	11.20% (216/1,928)
COVID-19 sporadic outbreaks in local city	
Yes	48.60% (937/1,928)
No	51.40% (991/1,928)
DASS-21 depression score [median (IQR)]	4.00 (0.00 10.00)
DASS-21 anxiety score [median (IQR)]	4.00 (0.00 10.00)
DASS-21 stress score [median (IQR)]	6.00 (0.00 12.00)
Quality of sleep^a^	
Poor	45.07% (869/1,928)
Good	54.93% (1,059/1,928)

IQR, Interquartile range; COVID-19, Corona virus disease 2019; DASS-21, Depression anxiety stress scales 21; PSQI, Pittsburgh sleep quality index.

^a^Indicates that poor sleep quality was defined as participants with a total PSQI score of six points or above.

### Development of the model

**FIGURE 2:**
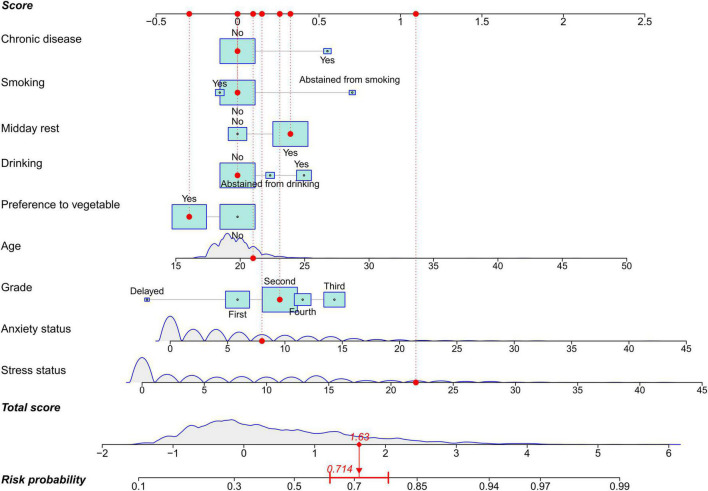
A nomogram to predict sleep quality among university students. The nomogram is comprised of nine features and three axes (score, total score, and risk probability axes). Each feature is able to obtain a score by referring to the score axis, the total score is the sum points of the nine features, and participant’s risk probability can be calculated by drawing a line downward from the total score axis to the risk probability axis. In the nomogram, quantitative features are depicted as density curves to visualize distribution, and qualitative features including age, anxiety, and stress are presented as boxes. The size of boxes indicates proportions in each feature.

Multivariate analysis showed that older age (*P* = 0.006), a higher grade (*P* = 0.024), previous smoking (*P* = 0.038), drinking (*P* = 0.017), midday rest (*P* = 0.013), chronic disease (*P* = 0.019), anxiety (*P* = 0.001), and stress (*P* < 0.001) were significantly associated with a higher rate of poor sleep quality (Table 2), while preference for vegetables (*P* = 0.005) was significantly associated with better sleep quality. Thus, these nine features were all included to develop the prediction model. To promote the application of the model, a nomogram was created to evaluate the probability of poor sleep quality (Figure 2). An example of how to use the nomogram was shown as follows. A 21-year-old university student who was in the second year of school did not have a chronic disease and was not a current smoker or drinker. This student had a habit of midday rest and preferred eating vegetables, and the Student’s anxiety and stress scores were 8 and 22, respectively. Each feature could obtain a score by referring to the score axis, and the total score (1.63) was the combination of the nine features. By drawing a line downward to the risk probability axis, users could obtain the predicted probability of poor sleep quality (71.40%) and its 95% CI.

**TABLE 2:** Multivariate analysis of characteristics for predicting poor sleep quality among university students in the training group.

Characteristics	Patients (*n* = 1,555, %)	OR	95% CI		*P*-value
			LL	UL	
(Intercept)		0.015	0.003	0.090	<0.001
Gender					
Male	632 (40.64%)	Reference			
Female	923 (59.36%)	1.301	0.988	1.711	0.061
Age [median (IQR), years]	19.00 (19.00, 20.00)	1.129	1.035	1.231	0.006
Grade					
First year	351 (22.57%)	Reference			
Second year	750 (48.23%)	1.205	0.883	1.644	0.239
Third year	271 (17.43%)	1.612	1.066	2.439	0.024
Fourth year	170 (10.93%)	1.236	0.730	2.094	0.431
Delayed graduation	13 (0.84%)	0.828	0.158	4.347	0.824
Marital status					
Single	1,187 (76.33%)	Reference			
Dating	356 (22.89%)	0.857	0.646	1.137	0.285
Married	12 (0.77%)	0.095	0.008	1.160	0.065
Smoking					
No	1,422 (91.45%)	Reference			
Abstain from smoking	45 (2.89%)	2.148	1.042	4.429	0.038
Yes	88 (5.66%)	0.997	0.567	1.753	0.991
Drinking					
No	1,256 (80.77%)	Reference			
Abstain from drinking	76 (4.89%)	1.433	0.820	2.505	0.206
Yes	223 (14.34%)	1.562	1.085	2.250	0.017
Having a habit of midday rest					
No	340 (21.86%)	Reference			
Yes	1,215 (78.14%)	1.443	1.080	1.928	0.013
Monthly expense (¥)					
<2,000	1,177 (75.69%)	Reference			
≧2,000 and < 5,000	363 (23.34%)	1.193	0.907	1.568	0.207
≧5,000 and < 10,000	8 (0.51%)	0.929	0.134	6.436	0.941
≧10,000	7 (0.45%)	1.020	0.120	8.634	0.986
Chronic disease					
No	1,487 (95.63%)	Reference			
Yes	68 (4.37%)	1.983	1.118	3.518	0.019
Preference to low salt and fat food					
No	1,086 (69.84%)	Reference			
Yes	469 (30.16%)	0.912	0.696	1.197	0.508
Preference to oil food					
No	1,153 (74.15%)	Reference			
Yes	402 (25.85%)	0.846	0.641	1.116	0.237
Preference to barbecue					
No	1,112 (71.51%)	Reference			
Yes	443 (28.49%)	1.258	0.960	1.650	0.097
Preference to red meat					
No	504 (32.41%)	Reference			
Yes	1,051 (67.59%)	1.011	0.782	1.306	0.935
Preference to vegetable					
No	793 (51.00%)	Reference			
Yes	762 (49.00%)	0.704	0.551	0.900	0.005
Preference to fruit					
No	636 (40.90%)	Reference			
Yes	919 (59.10%)	1.199	0.929	1.546	0.163
Sedentary time (hours)					
<1	81 (5.21%)	Reference			
≧1 and < 3	302 (19.42%)	0.731	0.409	1.305	0.289
≧3 and < 6	527 (33.89%)	0.747	0.428	1.305	0.305
≧6	645 (41.48%)	0.831	0.476	1.449	0.514
Frequency of sports per week					
0	366 (23.54%)	Reference			
1−2	615 (39.55%)	1.166	0.723	1.880	0.530
3−4	334 (21.48%)	1.371	0.841	2.236	0.206
≧5	240 (15.43%)	0.973	0.582	1.626	0.917
Sport type					
None	366 (23.54%)	Reference			
Aerobic exercise	655 (42.12%)	0.991	0.659	1.491	0.967
A middle between aerobic and anaerobic exercise	355 (22.83%)	1.153	0.755	1.761	0.511
Anaerobic exercise	179 (11.51%)	Not applicable			
COVID-19 sporadic outbreaks in local city					
No	646 (41.54%)	Reference			
Yes	909 (58.46%)	0.910	0.713	1.161	0.447
DASS depression score [median (IQR)]	4.00 (0.00 10.00)	1.018	0.989	1.049	0.223
DASS anxiety score [median (IQR)]	4.00 (0.00 10.00)	1.064	1.024	1.104	0.001
DASS stress score [median (IQR)]	6.00 (0.00 12.00)	1.071	1.038	1.104	<0.001

OR, odds ratio; CI, confident interval; LL, lower limit; UL, upper limit; IQR, interquartile range; COVID-19, coronavirus disease 2019; DASS-21, Depression Anxiety Stress Scale 21.

### Predictive effectiveness of the model

**FIGURE 3:**
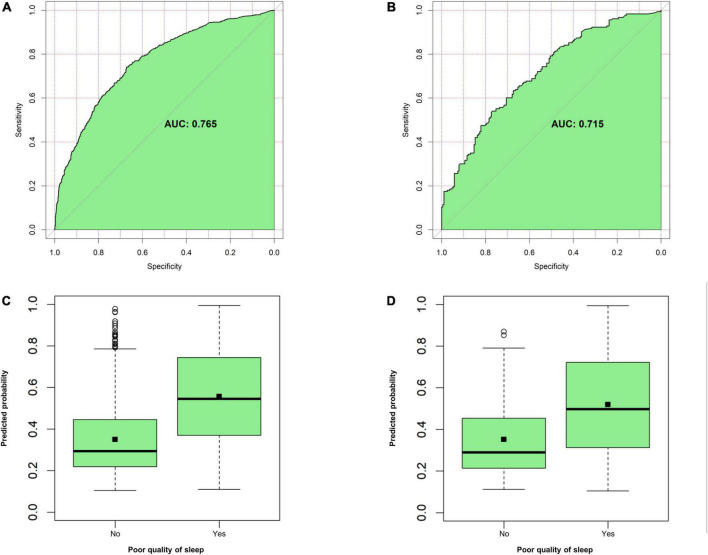
Evaluation of model’s discrimination. **(A)** Area under the curve (AUC) for the model in the training group. **(B)** AUC for the model in the validation group. **(C)** Discrimination slope for the model in the training group (0.207, *P* < 0.001). **(D)** Discrimination slope for the model in the validation group (0.167, *P* < 0.001). A discriminative plot is plotted with an actual event (yes vs. no) against a predicted probability of poor sleep quality. Discrimination slope is the mean difference of predicted probabilities between participants with actual poor sleep quality and those without it.

**FIGURE 4:**
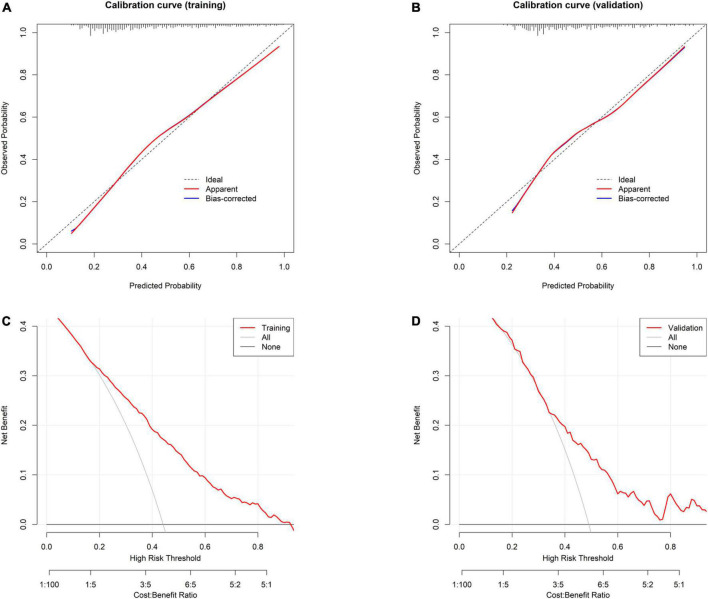
Evaluation of model’s calibration and clinical usefulness. **(A)** Calibration curve for the model in the training group. **(B)** Calibration curve for the model in the validation group. Calibration curve is plotted with predicted probability against observed probability. A dotted diagonal line in the curve indicates perfect consistency between the predicted and observed probability. **(C)** Decision curve analysis for the model in the training group. **(D)** Decision curve analysis for the model in the validation group. Decision curve is plotted with different thresholds against net benefit. Larger space between the red line and the two reference (a treat-for-all line and a treat-for-none line) lines indicates better clinical usefulness.

The AUC of the model was 0.765 (95% CI: 0.742–0.789) in the training group (Figure 3A) and 0.715 (95% CI: 0.664–0.766) in the validation group (Figure 3B), and the corresponding discrimination slopes were 0.207 (Figure 3C) and 0.167 (Figure 3D). Models’ accuracy, sensitivity, specificity, NPV, PPV, and other metrics are summarized in Table 3. Calibration curves demonstrated good consistency between predicted and observed probability in both training (Figure 4A) and validation (Figure 4B) groups, indicating excellent calibrating ability of the model. Decision curve analysis also showed favorable clinical usefulness in the training (Figure 4C) and validation (Figure 4D) groups.

**TABLE 3:** Predictive effectiveness of the model to predict risk probability of poor sleep quality among university students.

Prediction measures	Training group	Validation group
Brier score	0.195	0.221
Brier_scaled_ score	0.208	0.115
AUC (95% CI)	0.765 (0.742–0.789)	0.715 (0.664–0.766)
Discrimination slope	0.207	0.167
Accuracy	0.703	0.657
Threshold	0.378	0.475
Specificity	0.673	0.768
Sensitivity	0.741	0.541
NPV	0.767	0.635
PPV	0.641	0.692
Precision	0.641	0.692
Recall	0.741	0.541
Youden	1.414	1.309

AUC, Are under the curve; CI, Confident interval; NPV, Negative predictive value; PPV, Positive predictive value.

### Risk classification based on the model

The optimal cutoff value (43.00%) was obtained by calculating the round mean threshold of the training (37.80%) and validation (47.50%) groups. Therefore, this study defined that participants who had a predicted probability of 43.00% or above belonged to the high-risk group, while participants who had a predicted probability of less than 43.00% belonged to the low-risk group (Table 4). Based on the optimal cutoff value, the actual probability of poor sleep quality was 29.03% (317/1,092) in the low-risk group and 66.03% (552/836) in the high-risk group (*P* < 0.001), which indicated that participants in the low-risk group were 2.27 times more at risk of developing poor sleep quality than those in the high-risk group.

**TABLE 4:** Risk classification based on the nomogram and corresponding predicted and actual probability of poor sleep quality among the entire cohort of university students.

Groups	Patients (*n* = 1,928)	Probability of poor sleep quality		*P*-value^a^
		Predicted	Actual	
Low-risk (<43%)	1,092	27.09%	29.03% (317/1,092)	<0.001
High-risk (≧43%)	836	66.05%	66.03% (552/836)	

^a^Indicates the P-value was calculated from the Chi-square test after a comparison between the low-risk and high-risk groups in the actual probability of poor sleep quality.

## Discussion

Sleep problems are common among university students. In this study, we found that the incidence of poor sleep quality was up to 45.07%, and this number was consistent with other studies (2–5). Literature reported that 31.00–65.00% of university students suffered from poor sleep quality (2–5). To address this issue, it is necessary to accurately predict the probability of poor sleep quality, and thus appropriate interventions could be timely administered accordingly. This study successfully proposed a prediction model to evaluate the probability of poor sleep quality after analyzing 1,928 college students. A nomogram was created to promote the application of the prediction model. The prediction model had favorable predictive effectiveness in terms of calibration and discrimination.

This model was comprised of nine features, including age, grade, smoking, drinking, midday rest, chronic disease, anxiety, and stress, which were risk factors for poor sleep quality, and preference to vegetables, which was a protective factor for sleep quality. The results were in line with the currently available literature (2, 10–12, 25). For example, Xu et al. (10) reported that a high grade level, living in rural areas, depression, and anxiety might be negative impactors of poor sleep quality. Almojali et al. (12) found that a high level of stress and poor sleep quality were statistically correlated after analyzing 263 university students. Wang et al. (11) revealed that less exercise, skipping breakfast, and a higher grade were relevant to poor sleep quality. Li et al. (2) showed that for young people, alcohol use, gambling behaviors, and less frequency of sports per week were significant predictors of poor sleep quality. Alghwiri et al. (25) showed that pain and other systematic diseases were risk factors for predicting poor sleep quality after analyzing 1,600 university students.

In addition, the prediction model is gradually applied to evaluate sleep quality. For example, Chen et al. (16) developed the rapid classification scale for sleep quality to screen and subgroup poor sleep quality using four main variables, including sleep quality, hypnotic use, sleep onset, and lacking enthusiasm. But Chen’s scale was designed specifically for older adults, and the young were not enrolled, which meant that this scale was not applicable to the young population. Sai et al. (17) investigated insomnia-related factors and further constructed a prediction nomogram to identify patients with insomnia in an early stage according to a cross-sectional survey and an analysis of 1,133 participants. These participants were rescuers working in a military unit. Zhou et al. (26) proposed a novel framework to predict the quality of sleep according to the dynamic functional network connectivity after analyzing the fMRI data from human connectome project (HCP). Sadeghi et al. (18) achieved the sleep quality prediction with the help of physiological signals, including heart rate, electrodermal activity, body movement, and skin temperature, all of which were collected from a wearable device. The accuracy was about 75% for sleep quality among caregivers of dementia patients. The above scales generally had good accuracy, but the availability of information and population differences might compromise their applications to ordinary university students. This study included nine parameters, which were easily available, especially for university students. To the best of our knowledge, this model was the first to predict the poor sleep quality, especially among university students. This model was presented in the format of nomogram. In addition, risk stratification was achieved in the study, and it showed that participants in the low-risk group were 2.27 times more at risk of developing poor sleep quality than those in the high-risk group. Thus, more attention should be paid to participants among the high-risk group.

What can we do for participants in the high-risk group? On conducting the survey, COVID-19 is still prevailing. The great pandemic could affect the quality of sleep among university students due to lockdown, social distance, and stay-at-home orders, possibly because the negative mental health status that was aroused by the great pandemic was a significant contributor to poor sleep quality (27, 28). Luckily, with the normalization of preventing and controlling the great pandemic, its impact on sleep quality might be decreasing among university students. As depicted in this study, the feature (COVID-19 sporadic outbreaks in the participant’s city) did not show any statistically significant association with sleep quality. But, considering the unexpected length and severity of the outbreaks, intervention measures to deal with the psychological health of university students were still warranted since good mental health status was remarkably beneficial to sleep quality. Furthermore, researchers also pointed out that some measures might be effective to boost sleep quality, such as predefined sports programs, sleep education programs, interesting entertainment, and avoiding the overuse of mobile phones (15). But a systematic review showed that the effectiveness of sleep education on sleep behavior and sleep quality needed more supportive evidence (29). Therefore, future studies on the approaches and contents of sleep education program are still needed. Regarding Chinese traditional medicine, a traditional herb, rosemary, might be helpful to reduce mental distress and improve sleep quality among university students based on a randomized clinical trial after analyzing 68 participants (30). This study further demonstrated that some measures to quit smoking and drinking, eat more vegetables, avoid midday rest, treat chronic disease, and alleviate anxiety and stress moods would also be great helpful to sleep quality.

### Limitations

This study had certain limitations. First, this is a cross-sectional study in nature, and thus causalities between variables were unknown, although associations between variables could be analyzed in the study. Therefore, it needs further investigation on the causalities between quality of sleep and mental health. But we speculated that those variables can mutually affect each other. In other words, poor sleep quality can contribute to anxiety and depression and vice versa. Second, some variables, such as smartphone use severity (14, 15), were not taken into account when we initially designed this study, but it is relatively difficult to evaluate this variable due to no recognized criteria. Third, this model was not widely validated in other centers, despite favorable predictive effectiveness of the model.

## Conclusion

Sleep quality is far from satisfactory among university students. The study develops a prediction model that can be a helpful tool to stratify sleep quality, especially among university students. More attention needs to be paid to participants in the high-risk group. Some intervention measures or preventive strategies to quit smoking and drinking, eat more vegetables, avoid midday rest, treat chronic disease, and alleviate anxiety and stress may be considerably beneficial in improving sleep quality.

## Data availability statement

The raw data supporting the conclusions of this study will be made available by the authors, without undue reservation.

## Ethics statement

This study was approved by the Academic Committee and Ethics Board of the Xiamen University of Technology (No. 202001). The patients/participants provided their informed consent to participate in this study.

## Author contributions

LRZ, HZ, and MY: study design. LRZ, HZ, MY, YZ, and GC: data collection, analysis, and interpretation. LRZ, HZ, MY, CL, and LZ: drafting of the manuscript. All authors: critical revision of the manuscript and approval of the final version for publication.
